# Sedation Monitoring and Management during Percutaneous Endoscopic Lumbar Discectomy

**DOI:** 10.1155/2016/3931567

**Published:** 2016-05-19

**Authors:** Menekse Oksar, Tulin Gumus, Orhan Kanbak

**Affiliations:** ^1^Anesthesiology and Reanimation Department, Mustafa Kemal University Medical School, 31100 Hatay, Turkey; ^2^Anesthesiology and Reanimation Department, Ankara Ataturk Training and Research Hospital, 06800 Ankara, Turkey

## Abstract

Percutaneous endoscopic laser discectomy (PELD) is a painful intervention that requires deep sedation and analgesia. However, sedation should be light at some point because cooperation by the patient during the procedure is required for successful surgical treatment. Light sedation poses a problem for endotracheal intubation, while patients placed in the prone position during percutaneous endoscopic discectomy pose a problem for airway management. Therefore, under these conditions, sedation should be not deeper than required. Here we report the sedation management of three cases that underwent PELD, with a focus on deep and safe sedation that was monitored using bispectral index score and observer's assessment of alertness/sedation score.

## 1. Introduction

Although a bispectral index score (BIS) of 70 and an observer's assessment of alertness/sedation (OAA/S) score of 3 are commonly used to assess adequate sedation during anesthesia, their use has not been described for patients placed in the prone position during painful procedures. A combination of OAA/S scores and an electroencephalography- (EEG-) derived monitoring tool during sedation titration can be helpful in avoiding oversedation without verbal stimulation in these cases. Here we report the sedation management of three cases that underwent PELD, with a focus on deep and safe sedation that was monitored by BIS and OAA/S score. Preoperative assessments were performed, and written consent was preoperatively obtained from each patient. In the operating room, routine electrocardiogram, noninvasive blood pressure, peripheral capillary oxygen saturation, OAA/S score, and BIS were monitored, and 2 mg midazolam and 100 *μ*g fentanyl were preoperatively administered. Sedation was initiated with 1 mg propofol per kg body weight as an intravenous bolus injection. Subsequently, sedation was maintained with 10 mg/mL propofol and 40 *μ*g/mL remifentanil infusions through an automated infusion system (Perfusor® compact S; B. Braun Melsungen AG, Melsungen, Germany). The initial infusion rates were high; however, they were decreased over the course of the procedure (propofol was reduced from 3 to 2 mg/kg/h and remifentanil from 0.125 to 0.0625 *μ*g/kg/min) by primarily considering the target BIS or OAA/S score and keeping the patients immobile during the procedure and allowing them to interact with the surgeon as required. The infusion rates of propofol and remifentanil during the entire procedure and their effects on spontaneous breathing were recorded (Table 1). Adequate and safe sedation was achieved by monitoring a steady BIS of 60–70 and titrating sedative doses during the interval prior to the painful surgical intervention. However, during the painful surgical intervention, the clinical sedation status might change, and it was the primary target to achieve a OAA/S score of 3 to enable comfortable surgical intervention. Hence, we primarily targeted a specific sedation state and subsequently monitored and adjusted this state during the procedure, considering the requirements for sedation and/or analgesia at any particular time.

## 2. Case Presentation

*Case  1*. A 33-year-old male, with American Society of Anesthesiologists (ASA) physical status I, was scheduled for PELD. The patient was monitored, and sedoanalgesia was achieved as described above. The landmarks of the target vertebra and skin window location, which were calculated from the disc inclination line at the lateral projection, were determined using the c-arm technique. After the skin window was selected, the subcutaneous tissue and trajectory tract were infiltrated at the L5-S1 level with 1% lidocaine. An 18-gauge needle was used to start PELD using the previously described posterolateral approach [1]. During the intervention, when instantaneous deep sedation was required, additional doses of propofol were administered during painful spinal neuroendoscopy because of an annular fenestration. The patient's breathing was noninvasively supported using a face mask because of respiratory depression. 

*Case  2*. A 64-year-old male, with ASA physical status II, had a recurrent lumbar disc hernia and was scheduled for PELD. Monitorization, premedication, sedoanalgesic protocol, and intervention were similar to those for Case  1. Although the targeted sedation depth was achieved, an additional bolus dose of propofol was required during instrumentation. Respiratory depression was observed, and positive pressure ventilation was administered.

*Case  3*. A 63-year-old male, with ASA physical status I, was scheduled for PELD. Monitorization, premedication, and bolus dose of propofol were similar to those for Cases  1 and 2. However, sedation maintenance was performed using propofol until spinal instrumentation because the target BIS and OAA/S score had already been achieved. Remifentanil was administered immediately prior to spinal instrumentation and local anesthetic injection. An additional bolus dose of propofol was not required during the procedure. Respiratory depression was observed for such a short period that very little ventilatory assistance was required (Table 1).

## 3. Discussion

BIS is used as an EEG-derived parameter for intraoperative monitoring of anesthetic depth [2] and correlates with the sedation level and loss of consciousness during administration of propofol, midazolam, isoflurane, or alfentanil [3]. Bagchi et al. found a difference in the time taken to achieve BIS of 70 and an OAA/S score of 3 during the onset of sedation with propofol and midazolam [4]. However, the same authors revealed that the time taken to achieve BIS of 90 was the same as that to achieve an OAA/S score of 5 during recovery from sedation [5]. A moderate-to-strong correlation between BIS and OAA/S score during sedation onset and a strong correlation during arousal from propofol sedation were also reported.

In this report, using both BIS and OAA/S score as monitoring tools to achieve the target sedation depth, we aimed to avoid administering excessive drugs to protect the spontaneous breathing of patients. As the patients became apneic before and during spinal intervention, we reviewed the surgical requirements and revised the methods to provide a satisfactory and safe sedation depth and analgesia level for Case  3. Depression in spontaneous breathing was observed during the combined infusion of propofol and remifentanil or propofol alone. Therefore, in these cases, the actual reason for assisted ventilation of patients was respiratory depression because of the sedative agent alone or in combination with remifentanil. According to these methods, our primary concern was to separately consider the sedative and analgesic indications according to the surgical requirements, reduce sedative doses, and limit the use of opioid analgesics with the interventional process when we attempted to achieve the target sedation depth using dual monitoring, which should be considered in painful procedures and in patients placed in the prone position when endotracheal intubation is not desired. The practical advantage of this method is that it provides all conditions for intervention such as immobilization, avoidance of drug excess with adequate sedation depth, and cooperation by the patient during unpainful periods. Although the data provided in the present report are limited, respiratory depression in patients in the prone position suggests that local anesthesia and sedoanalgesic administration under dual monitoring using BIS or OAA/S score, regarding the painful or unpainful periods, should be the primary focus during PELD. This will help in optimizing sedation depth, which may not interfere with the spontaneous breathing of the patient, thereby keeping the patient safe.

## Figures and Tables

**Table 1:** Infusion rates of propofol (10 mg/mL) and remifentanil (40 *μ*g/mL) used in sedation maintenance and spontaneous breathing during the PELD procedures.

	Propofol (mg/kg/h)			Remifentanil (*μ*g/kg/min)			Spontaneous breathing (+, Yes; −, No)		
	Case 1	Case 2	Case 3	Case 1	Case 2	Case 3	Case 1	Case 2	Case 3
T1	0	0	0	0	0	0	+	+	+
T2A	3	2	1.3	0.125	0.125	0	−	−	+
T2B	2	2	0.8	0.0625	0.0625	0	+	+	+
T3A	1	3	1.7	0.0625	0	0.138	+	+	+
T3B	1	1	1.7	0	0	0.138	−	+	−
T4	0	0	0	0.0625	0.0625	0	+	+	+

T1: at the beginning; T2A: after IV propofol bolus; T2B: after reducing propofol and remifentanil to obtain targeted BIS and SS scores; T3A: at the beginning of the spinal neuroendoscopy and discectomy; T3B: after extra doses of propofol during the spinal neuroendoscopy and discectomy; T4: at the end of surgical procedure.

## References

[1] Yeung A. T., Tsou P. M. (2002). Posterolateral endoscopic excision for lumbar disc herniation: surgical technique, outcome, and complications in 307 consecutive cases. *Spine*.

[2] Sigl J. C., Chamoun N. G. (1994). An introduction to bispectral analysis for the electroencephalogram. *Journal of Clinical Monitoring*.

[3] Glass P. S., Bloom M., Kearse L., Rosow C., Sebel P., Manberg P. (1997). Bispectral analysis measures sedation and memory effects of propofol, midazolam, isoflurane, and alfentanil in healthy volunteers. *Anesthesiology*.

[4] Bagchi D., Mandal M. C., Das S., Basu S. R., Sarkar S., Das J. (2013). Bispectral index score and observer's assessment of awareness/sedation score may manifest divergence during onset of sedation: Study with midazolam and Propofol. *Indian Journal of Anaesthesia*.

[5] Bagchi D., Mandal M. C., Basu S. R. (2014). Arousal time from sedation during spinal anaesthesia for elective infraumbilical surgeries: comparison between propofol and midazolam. *Indian Journal of Anaesthesia*.

